# Identification of Degenerative Cervical Myelopathy in the Chiropractic Office: Case Report and a Review of the Literature

**DOI:** 10.7759/cureus.30508

**Published:** 2022-10-20

**Authors:** Robert J Trager, Gabriel A Smith, Collin M Labak, Patrick J Battaglia, Jeffery A Dusek

**Affiliations:** 1 Chiropractic, University Hospitals Cleveland Medical Center, Cleveland, USA; 2 Chiropractic, Logan University, Chesterfield, USA; 3 Neurosurgery, University Hospitals Cleveland Medical Center, Cleveland, USA; 4 Health Policy and Interdisciplinary Care, Logan University, Integrated Health Centers, Chesterfield, USA; 5 Research, University Hospitals Cleveland Medical Center, Connor Whole Health, Cleveland, USA

**Keywords:** spinal manipulation, spinal fusion, spinal cord compression, differential diagnosis, chiropractic, cervical vertebrae

## Abstract

Degenerative cervical myelopathy (DCM) is a common cause of spinal cord dysfunction, yet it may be challenging to identify as it presents with variable symptoms.

A 62-year-old woman presented to a chiropractor with a three-month exacerbation of neck pain, hand/finger numbness, and torso dysesthesia. She had previously seen primary care, physical therapy, rheumatology, and pain management. Previous cervical magnetic resonance imaging showed moderate cervical canal stenosis; however, previous providers had diagnosed her with radiculopathy and possible carpal tunnel syndrome yet had not requested neurosurgical consultation. On examination, the chiropractor identified sensorimotor deficits, hyperreflexia, and bilateral Hoffman reflexes, and referred the patient to a neurosurgeon for suspected DCM. The neurosurgeon performed an anterior cervical discectomy and fusion from C4-7. The patient’s symptoms and disability level improved within two months of follow-up. We identified 11 previous cases in which a chiropractor suspected DCM which was then confirmed by a surgeon. Including the current case (i.e., 12 total), patients were older and mostly male; 50% had neck pain, 92% had hyperreflexia. Chiropractors referred each patient to a surgeon; 83% underwent cervical spine surgery.

This case highlights the identification of DCM by a chiropractor and referral for neurosurgical evaluation with a positive outcome. Patients with previously undiagnosed DCM may present to chiropractors with varied symptoms and examination findings. DCM may contraindicate spinal manipulation and instead warrant surgery. Accordingly, chiropractors play a key role in the detection and referral of patients with misdiagnosed or overlooked DCM.

## Introduction

Degenerative cervical myelopathy (DCM) is progressive cervical spinal cord dysfunction caused by the narrowing of the spinal canal [1]. Affecting approximately 2.3% of individuals above age 16 [2], DCM is the most common cause of spinal cord dysfunction worldwide [3-5] and classically presents with subjective ataxia, coordination loss, weakness, and sensory changes [6]. There is a need for musculoskeletal therapists such as chiropractors to recognize this condition early in its course [6], as treatment with spinal manipulation may exacerbate symptoms [7,8], and delayed detection of DCM reduces the likelihood of improvement with surgery [5,9].

DCM will become more prevalent as the global population becomes older [10]. While congenital cervical spinal stenosis and smoking are risk factors for DCM, there is a limited understanding of other contributing factors [3,11]. Diagnostic delay is common, with a mean interval from symptom onset to diagnosis of DCM of 2.2 ± 2.3 years [12]. Providers make the diagnosis clinically as well as by correlating MRI findings of spinal stenosis and electrodiagnostic (e.g., electromyography) results [11].

Chiropractors are portal-of-entry healthcare providers who commonly manage neuromusculoskeletal complaints, chiefly those of the spine [13]. It is not clear how often chiropractors encounter patients with undiagnosed DCM [14]; however, about 60% of chiropractic patients are at least 55 years old [13], which suggests chiropractors may commonly encounter DCM, given its higher prevalence in older individuals. However, research regarding chiropractic and DCM is limited. In some previous case reports, the chiropractor identified DCM and referred the patient to a surgeon [15,16]. However, in other cases, the chiropractor did not suspect underlying DCM, which was then aggravated by cervical spinal manipulative therapy [7,8], a common treatment utilized by chiropractors.

Surgical decompression with or without fusion is the principal treatment used for moderate to severe DCM [17]. While there is limited research regarding non-surgical treatments for DCM, physical therapy, cervical traction, cervical orthoses, spinal injections, and close observation may be appropriate for patients with mild DCM [18]. In one recent prospective study, patients with DCM showed improvements in pain and disability after a program including massage, manual therapies, and exercises [19]. In another case series, patients with cervical canal encroachment, but not acute myelopathy or myelomalacia, showed improvements with cervical spinal manipulation [20].

Considering little research has examined the management of DCM in the chiropractic setting, and the importance of recognizing DCM to help avoid adverse treatment responses, we present a case in which a chiropractor suspected DCM in a patient and referred her to a spinal surgeon, prompting cervical fusion surgery for severe myelopathy.

## Case presentation

Patient information

**Figure 1:**
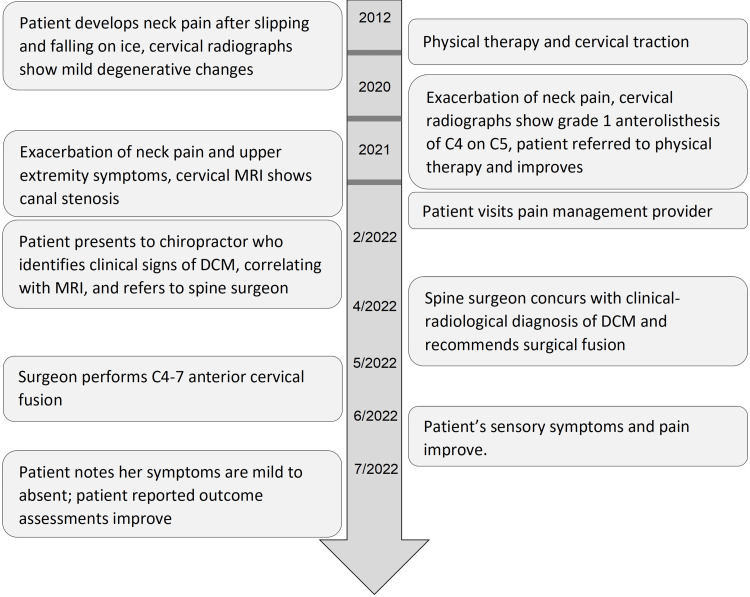
Timeline of care DCM - degenerative cervical myelopathy, MRI - magnetic resonance imaging Gray horizontal bars indicate breaks in the timeline between years before the timeline enters a month-by-month format.

**Figure 2:**
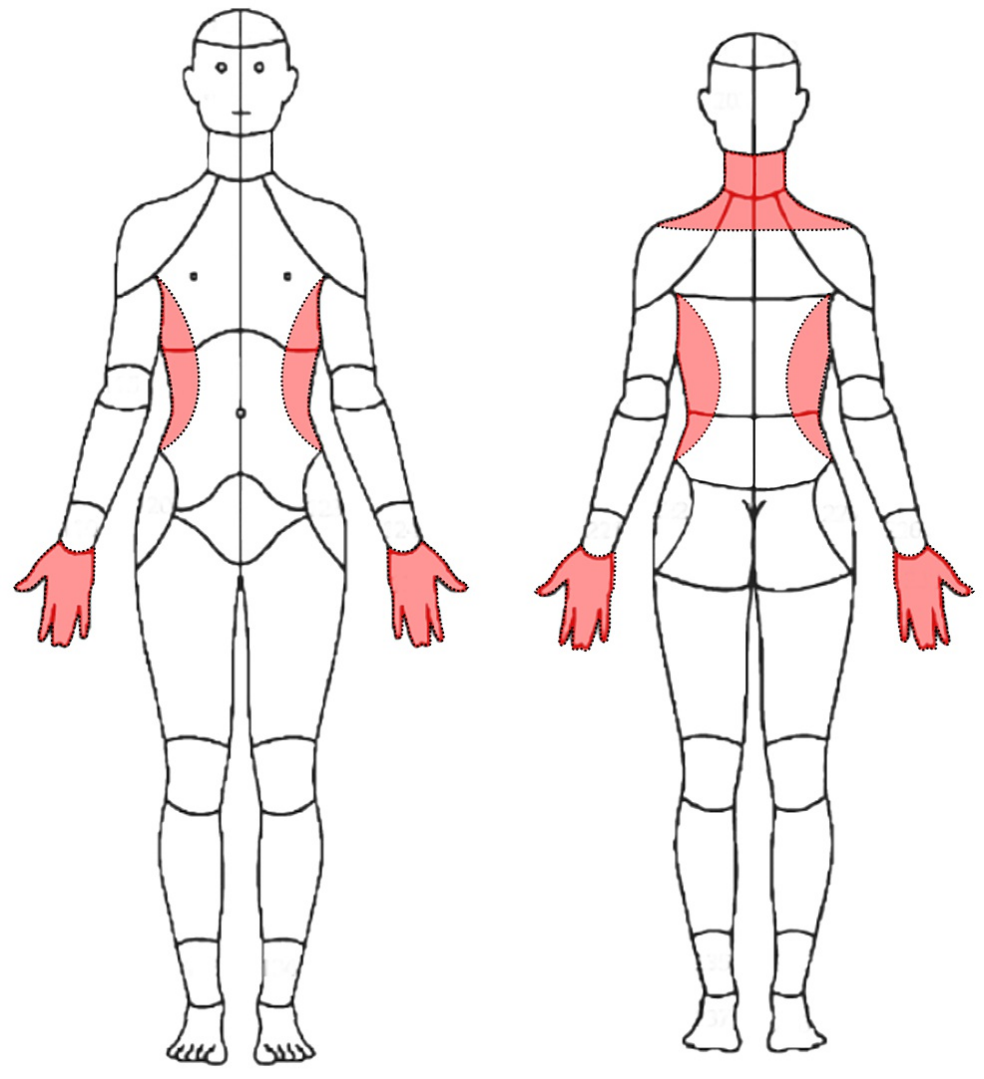
Patient's symptom distribution shown in shaded areas (red) The patient reported numbness and tingling in her hands bilaterally, including all fingers, neck pain, and warm dysesthesias along the torso bilaterally. Image is the Collaborative Health Outcomes Information Registry (CHOIR) body map, Creative Commons License from Alter et al. [21], modified by RT using GNU Image Manipulation Program (GIMP) version 2.10.30 to remove other labels and show the patient's pain distribution.

A 62-year-old female nonsmoker with a past medical history of asthma, hypertension, and bilateral carpal tunnel syndrome status post bilateral surgical decompression presented to a chiropractor in an integrative setting with a three-month exacerbation of frequent neck pain and tightness, with constant numbness and tingling in her hands diffusely and bilaterally (Figure 1). She rated her current neck pain 3/10 on the numeric rating scale with occasional severe pain at 8/10. She also noted a sensation of warmth along the lateral torso in the region inferior to the axilla extending to the iliac crest (Figure 2). The patient noted that it was hard to hold a pencil and tie her shoes, she dropped things frequently, and had difficulty performing tasks at work due to weakness of her hands. She also reported a reduction in sleep to less than six hours due to her symptoms. She denied any difficulties with walking or bowel or bladder disturbances.

**Figure 3:**
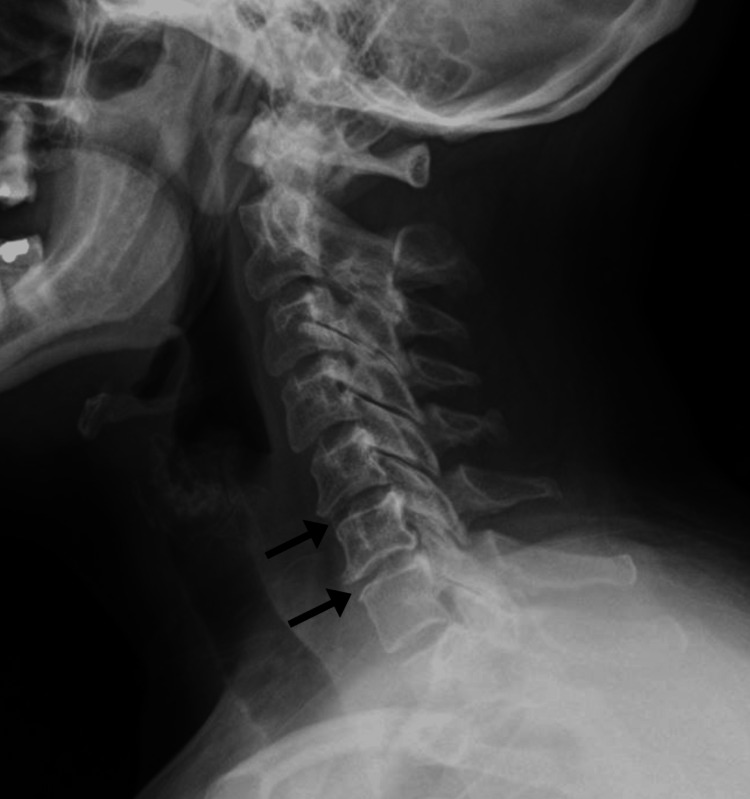
Cervical spine radiograph, lateral view, from 10 years prior to presentation. The radiograph shows reduction in disc spacing at C5-6 and C6-7 with marginal anterior osteophyte formation consistent with spondylosis (arrows). In other views (not shown), uncovertebral joint hypertrophy was evident on the right at C5-6 and on the left at C6-7 producing a mild degree of foraminal encroachment bilaterally.

Ten years prior to presentation to the chiropractor, she developed neck pain after she slipped and fell backward on ice. This prompted her to go to the emergency department, where she had radiographs of her cervical spine showing mild degenerative changes at C5-6 and C6-7 (Figure 3). Providers referred her to physical therapy, and according to her physical therapist's recommendation, she purchased a cervical traction device (Saunders) and used this regularly about a year until her symptoms improved.

**Figure 4:**
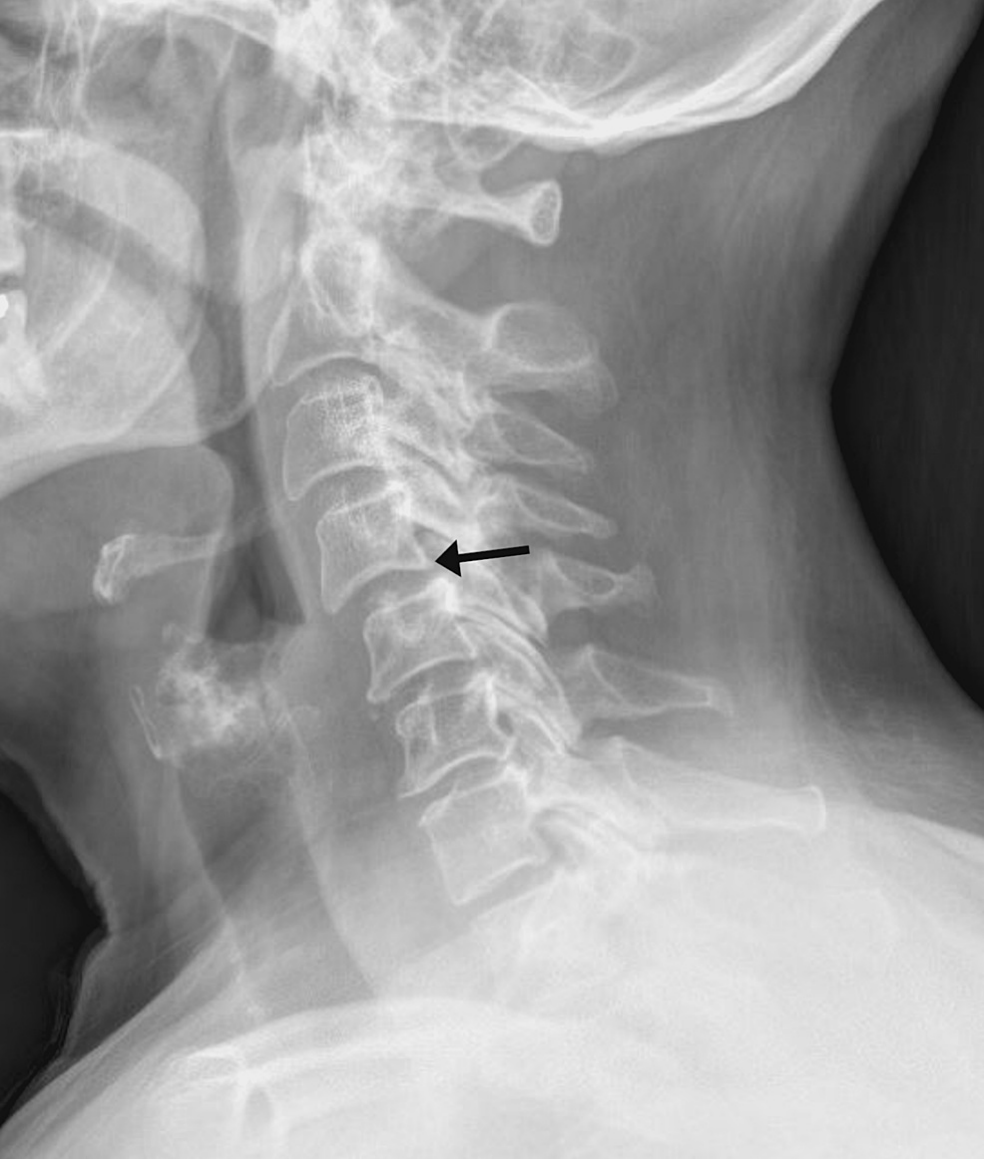
Cervical spine radiograph, lateral view, from 16 months before presentation There is a grade 1 anterolisthesis of C4 on C5 measuring 2.5 millimeters or 16% (arrow). Mild multilevel endplate sclerosis and osteophytosis is noted throughout the cervical spine, worst at C6-7.

Sixteen months prior to presentation she had an episode of moderate-to-severe left neck, shoulder, and arm pain, described as a generalized aching pain, which was keeping her up at night and causing her to become nauseous. She visited her primary care provider, who ordered cervical spine radiographs. These were notable for a grade 1 (i.e., less than 25%) degenerative anterolisthesis of C4 on C5, and multilevel spondylosis (Figure 4). Her primary care provider diagnosed her with cervical radiculopathy and again referred her for physical therapy. The patient also resumed using the cervical traction device at home. The patient reported improvement of her pain with physical therapy, yet noted ongoing numbness and tingling in the right upper extremity. After a brief period of relief, her symptoms fully returned.

**Figure 5:**
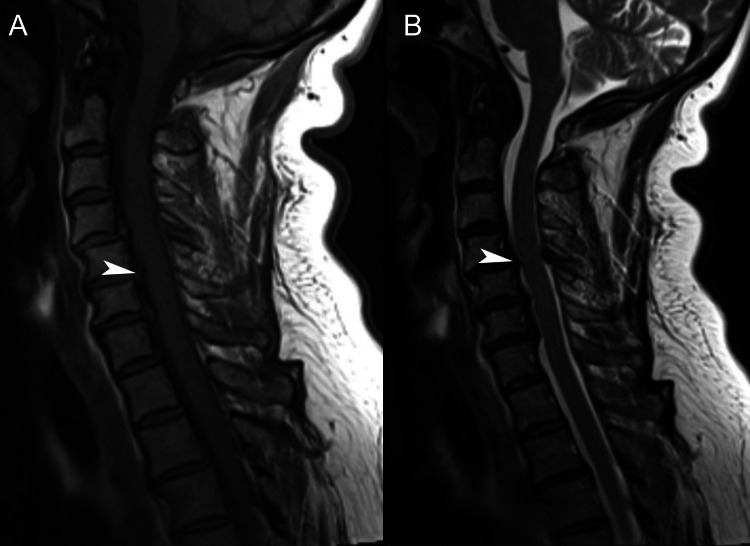
Cervical spine magnetic resonance imaging, sagittal views In the T1-weighted (A) and T2-weighted (B) views, moderate spinal canal stenosis secondary to facet/uncovertebral arthropathy and degenerative discogenic change is evident at C4-5 (arrowheads). Per Kang et al.'s classification [22], this stenosis is grade 2, considering there is >50% obliteration of the subarachnoid space and minimal deformation of the spinal cord. Additional mild spinal canal stenosis was noted at C5 through C7. The mid-sagittal canal diameter is 8 millimeters at C4-5 and 10 millimeters at C5-6 and C6-7. There is no abnormal signal change in the spinal cord.

Seven months prior to presentation, the patient presented to her primary care provider noting constant neck pain and radiating pain and numbness in the right upper extremity worsened with neck rotation. She noted neck pain that radiated into the right upper extremity when turning her neck to the right side. The primary care provider ordered a cervical spine MRI, the report for which noted moderate spinal canal stenosis from C5 through C7 (Figure 5), severe right foraminal narrowing at C5-6, and severe left neural foraminal narrowing at C6-7. The primary care provider diagnosed the patient with cervical radiculopathy and referred her to pain management.

Two months prior to presentation, the patient saw a pain management specialist who suspected the patient had cervical radiculopathy, secondary fibromyalgia, and had a recurrence of carpal tunnel syndrome. The pain management specialist performed an intramuscular injection of ketorolac tromethamine 60 mg, and later performed a bilateral interlaminar epidural steroid injection at C6-7. This provider also recommended coenzyme Q10 and B-complex vitamins. These therapies did not provide the patient with any relief, and the provider referred the patient to the chiropractor.

Clinical findings

On examination, the chiropractor noted that the patient had diminished active cervical spine range of motion in all planes with pain during movement and decreased sensation to light touch diffusely in her hands and fingers bilaterally. The patient had hypertonicity of the cervical paraspinal muscles and upper trapezius bilaterally. Her reflexes were 3+ diffusely and bilaterally, including the biceps, brachioradialis, triceps brachii, quadriceps, medial hamstring, and triceps surae. The Hoffman reflex was present bilaterally, while the Rossolimo sign and extensor plantar responses were absent. Muscle testing (Medical Research Council scale) revealed 3/5 strength of the extensor pollicis longus and finger abduction bilaterally and 4/5 strength of the biceps and triceps bilaterally.

The chiropractor considered that carpal tunnel syndrome or radiculopathy would not fully explain the patient's clinical features of neck pain, non-dermatomal upper extremity numbness, and weakness, hyperreflexia, and pathologic reflexes, and instead, DCM would be most consistent with these findings. Considering the patient had worsened over time and tried other forms of conservative care, including traction, physical therapy, and injections, the priority of management was to refer the patient to a spine surgeon.

The chiropractor informed the patient of this management plan, who expressed an understanding and voiced she had considered there was something more serious going on with her health. She further noted that she had already tried physical therapy and home traction for her neck pain over the past ten years and did not want to continue with any extensive therapies. She was, however, interested in a brief trial of gentle chiropractic treatments in the interim while she waited to see the spine surgeon.

The patient gave informed consent for a trial of three chiropractic sessions involving gentle soft tissue manipulation of the upper trapezius and dry needling with monofilament needles 0.25 millimeters (mm) by 40 mm in the cervical paraspinal and upper trapezius muscles, using ten needles total at each visit. This intended to reduce muscle hypertonicity and alleviate pain. Treatment also included spinal mobilizations performed in the mid-thoracic spine (T4-6) and thoracolumbar junction (T12-L2) to reduce spinal pain and improve mobility. The patient also resumed using her home cervical traction device. The patient tolerated chiropractic treatments well, which afforded her temporary relief of her neck pain; however, her other symptoms, including upper extremity numbness, weakness, and torso dysesthesias, were unchanged.

The patient visited the neurosurgeon two months after presenting to the chiropractor. On examination, this provider likewise identified sensory and motor deficits in the upper extremities, bilateral Hoffman reflex, and graded the quadriceps reflexes 4+. She rated her pain severity 8/10 on the visual analog scale; her Neck Disability Index was 56%, indicating severe disability; while her patient-derived version of the modified Japanese Orthopaedic Association (P-mJOA) score was an 11, suggestive of severe myelopathy. As the patient's clinical symptoms correlated with MRI findings of spinal canal stenosis, the neurosurgeon diagnosed the patient with DCM. Considering the patient had significant functional impairment with decreased ability to perform her normal activities of daily living, and had tried treatment options, including medications, formal physical therapy, injection, and chiropractic care, the neurosurgeon offered the option of C4-5 C5-6 and C6-7 anterior cervical discectomy and fusion with plating.

The neurosurgeon chose a ventral approach to the spine, given most of the patient's cord compression stemmed from intervertebral disc disease, which would be best addressed via an anterior approach. Additionally, an anterior approach favored the reduction of the C4-5 anterolisthesis.

One month later, three months after presenting to the chiropractor, the patient underwent cervical fusion surgery. The neurosurgeon completed a standard anterior approach to the spine, removed the posterior longitudinal ligament, and performed discectomies and bilateral foraminotomies at C4/5, C5/6, and C6/7, with the placement of six mm allograft interbody spacers. The neurosurgeon fixated a 46 mm anterior plate with 17 mm screws at each vertebra from C4-C7. There were no complications of surgery, and the patient was discharged the following day with a standard post-operative pain regimen.

**Figure 6:**
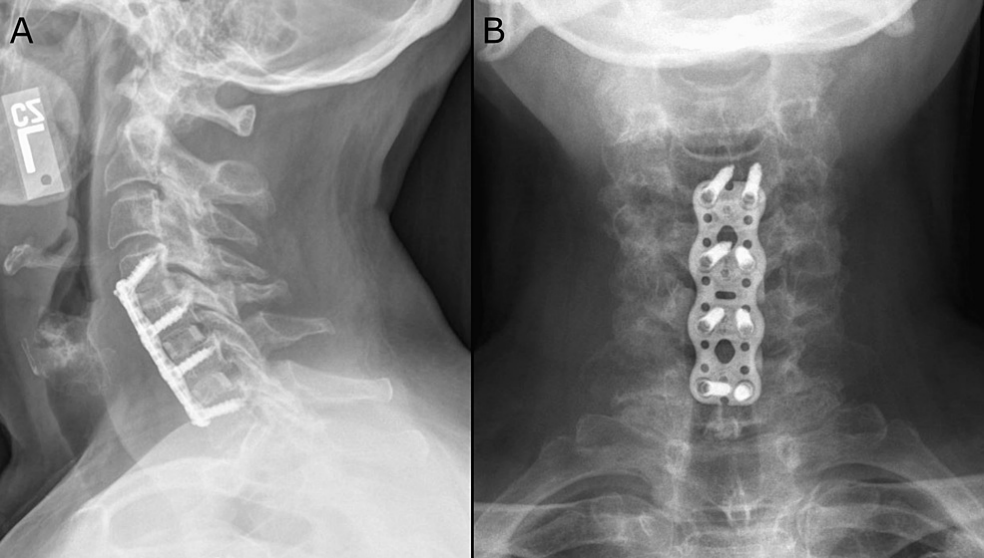
One-month post-surgery cervical spine radiographs. The lateral (A) and anteroposterior (B) views demonstrate successful anterior fusion from C4 to C7.

At her one-month post-operative visit, the patient reported an overall improvement in her right upper extremity paresthesias and overall activity. Post-operative radiograph revealed good placement of hardware (Figure 6). At her six-week post-operative visit, the patient reported her pain as 3/10 on the visual analog scale; her Neck Disability Index reduced to 16%, suggestive of mild disability; and her P-mJOA score increased to 15, suggestive of mild myelopathy.

Two months after surgery, the patient reported that she was doing much better and noted a reduction in all her symptoms. Her neck pain was now mild, and she noted the numbness in her hands had reduced to a faint tingling. The patient provided written consent for the publication of this case report and accompanying images.

## Discussion

This case illustrates a woman with neck pain and upper extremity symptoms with a working diagnosis of cervical radiculopathy and carpal tunnel syndrome and identified by a chiropractor as having clinical signs of DCM. Accordingly, the chiropractor referred the patient to a neurosurgeon, and she underwent an anterior cervical fusion with a reduction of pain and improvement of neurologic deficits following surgery.

The patient's symptoms, in this case, may have been multifactorial. Considering she had a decline in neurological function, hyperreflexia, and pathological reflexes, treatment of DCM was the priority. The patient may have had a radicular component to her symptoms, given the foraminal stenosis noted on the cervical spine MRI; however, the cervical spine surgery addressed this potential component. While the patient could have had a component of carpal tunnel syndrome, her entire-hand distribution was somewhat atypical for this diagnosis [23]. Recurrence of carpal tunnel syndrome after surgery is uncommon [24]. Conversely, bilateral carpal tunnel symptoms are suspicious for DCM [25], and the improvement of her hand deficits after cervical spine surgery also pointed to DCM as the source of her hand symptoms. Another interesting finding with limited research in this case is the patient's warm dysesthesia in the torso. According to one study, a girdle sensation around the trunk is associated with severe compression of the spinal cord and may localize to a lesion of the midline ventral cord in relation to the anterior spinal artery [26].

In the current case, the patient's previous MRI did not demonstrate signal intensity changes in the spinal cord; however, the combination of stenosis and clinical findings pointed to a diagnosis of DCM. Cord signal intensity changes, which are an inconsistent finding among patients with DCM [2,27], are associated with more permanent injury and a poorer response to surgery [5]. Accordingly, it is possible that the absence of cord signal intensity changes partly explains the patient's rapid improvement following surgery. Further, it is also possible that the patient's DCM was detected relatively early, thus yielding a positive surgical outcome [9]. Newer MRI techniques using diffusion tensor imaging are increasingly used as they have a greater ability to detect early spinal cord abnormalities in comparison to conventional MRI [28].

We searched the literature on September 23, 2022, to identify publications describing patients with previously undiagnosed DCM and whom a chiropractor suspected of having DCM. We excluded patients with previous surgery for DCM, or other causes of myelopathy (e.g., syringomyelia, transverse myelitis), and required patients to have advanced imaging of the cervical spine (i.e., MRI or computed tomography) supporting the clinical diagnosis of DCM. We searched PubMed, Google Scholar, and the Index to Chiropractic using the terms "myelopathy," "cervical stenosis," "myelomalacia," "chiropractic," and "chiropractor" and variations of these terms. Included articles and chiropractic review articles [29-31] were also hand-searched for references.

Our literature search identified 11 cases in which a chiropractor aided in the diagnosis of DCM [15,16,32-38]. In addition to the current case (i.e., total of 12 cases; Table 1), the mean patient age was 54 ± 14, with most patients being male (9/12, 75%). Neck pain was inconsistent, and cases reported this finding in 50% of patients. The most common examination abnormalities included hyperreflexia (11/12, 92%), Hoffman sign (7/12, 58%), clonus (5/12, 42%), and weakness, sensory deficit, and ataxia or balance deficit (each 4/12, 33%). Most patients subsequently underwent CT or MRI (9/12, 75%). Chiropractors referred all patients to a surgeon. Aside from two patients who the surgeon deemed ineligible for surgery, the remainder (10/12, 83%) underwent cervical spine surgery.

**Table 1:** Cases of degenerative cervical myelopathy identified by a chiropractor *Advanced imaging for the cervical spine including computed tomography or magnetic resonance imaging ACDF - anterior cervical discectomy and fusion, CTS - carpal tunnel syndrome, CT - computed tomography, MVC - motor vehicle collision, MRI - magnetic resonance imaging, PMR - physical medicine and rehabilitation, ROM - range of motion

Author(s), year	Patient age	Patient sex	Symptoms	Examination findings	Previous advanced imaging*	Management
Bolles et al., 2022 [30]	49	M	Chronic, atraumatic neck and arm pain, worsening gait/balance, hand dysesthesia, prior CTS surgery	Abnormal tandem walk, fasciculations, lower extremity hyperreflexia, apallesthesia, upper extremity weakness	No	Cervical MRI, referred to neurosurgeon, anterior decompression and disc replacement at C4/5, C5/6
Bolles et al., 2022 [30]	38	F	Neck pain after recent MVC; extremity paresthesia, unsteadiness	Abnormal tandem walk, hyperreflexia, Hoffman sign, clonus, upper and lower extremity weakness	No	Cervical MRI, referred to neurosurgeon, underwent ACDF
Cates and Soriano, 1995 [32]	55	M	Neck, shoulder and right arm pain and hand weakness	Hyperreflexia, upgoing Babinski, asymmetrical lower extremity sensory deficit	Yes	Referred to neurosurgeon, posterior decompressive laminectomy
Crawford et al., 1995 [33]	38	M	Neck stiffness, left shoulder and arm pain, finger numbness.	Sensory deficits, hyperreflexia, Hoffman sign, clonus, Lhermitte phenomenon	No	Radiographs, myelography, CT, referred to neurosurgeon, posterior decompressive laminectomy C5/6
Crawford et al., 1995 [33]	73	M	Low-back and right lower extremity pain	Ataxia, hyperreflexia, Hoffman sign	No	Radiographs, CT, referral to surgeon (not surgical candidate)
Current case, 2022	62	F	Neck, shoulder pain; hand numbness, weakness	Hyperreflexia, Hoffman sign, hand sensory deficit, upper limb weakness	Yes	Referral to neurosurgeon, ACDF C4-7
Boesch and Nekoomand, 2011 [34]	47	M	Low back pain and bilateral lower extremity weakness, nausea, dizziness, blurred vision	Unclear	No	Radiographs, MRI, surgical consultation; surgical decompression
King et al., 2011 [14]	53	F	Immobility of lower extremities, paresthesia of fingers, head, back	Hyperreflexia, Hoffman reflex, reduced C/S ROM	No	Cervical MRI, referred to neurosurgeon, underwent C3-7 posterior fusion
Murphy and Beres, 2008 [35]	38	M	Left arm pain, numbness, lower extremity paresthesias, weakness	Ataxia on tandem walking, increased sway with Romberg test, hyperreflexia, absent umbilical reflexes	Yes	Referred to neurosurgeon, ACDF
Price et al., 2021 [13]	58	M	Thoracic pain, dropping things	Hoffman reflex, hyperreflexia, ankle clonus, lower extremity weakness	No	Referred to PMR, MRI, C5/6, C6/7 ACDF
Toto, 1986 [36]	84	M	Hip pain, low back pain, constipation	Mixed hyper- and hyporeflexia; multiple pathological reflexes, including Hoffman sign; clonus, rigidity, sensory deficits	No	Cervical radiographs, CT, referred to neurologist, deemed not a surgical candidate
Troutner and Barbato, 2022 [31]	57	M	Neck pain, upper extremity paresthesia and weakness, CTS diagnosis	Abnormal tandem gait, hyperreflexia, clonus, Hoffman sign, hypoesthesia,	No	Cervical MRI, referred to neurosurgeon; C4-5 anterior cervical discectomy and fusion

The current case is like those previously published, where the patient underwent cervical spine surgery. The current case most resembles two of those previously published in which previous advanced imaging showed some degree of degenerative findings, yet a diagnosis of DCM had not been established [34,37]. Two of the previous cases also reported that the patient had a previous carpal tunnel syndrome diagnosis, as in the current case [32,33]. Another interesting finding is that almost half of the cases (i.e., 5/12) had a publication date of 2021 or later. It is unclear if this represents improving detection or awareness of DCM by chiropractors, or is a phenomenon related to greater involvement in publishing case reports.

When suspecting DCM, chiropractors should avoid any cervical spinal manipulation until this diagnosis is evaluated further, as this therapy could exacerbate DCM [7,8]. Chiropractors should not be reliant on the presence of neck pain to identify DCM, which was inconsistent among patients in this review and prior research on DCM in general [39]. Patients should undergo cervical MRI to characterize the degree of spinal canal stenosis and cord compression [6]. If imaging and examination findings correlate with DCM, chiropractors should refer patients to a spine surgeon promptly for additional evaluation as they may require surgery. There is limited evidence to suggest that mild cases of cervical stenosis may benefit from manual therapies such as those provided by a chiropractor [19,20]. Accordingly, for patients who are not surgical candidates or prefer to avoid surgery, clinicians could consider such therapies on a case-by-case basis after clearance by the surgical provider.

Strengths and limitations

Strengths of this case include its long observation window, insights from the chiropractor, surgical team, and radiologist, and the apparent benefit of integration of a chiropractor into a large healthcare organization. In the current case, the chiropractor played a vital role in the diagnosis of DCM, which was facilitated via access to the patient's previous imaging, imaging reports, and notes in the shared medical records system. Further, the chiropractor was able to make a customized referral note to a neurosurgeon within the same healthcare facility, which may have accelerated the process of obtaining surgery for DCM. Prompt communication of this information is vital to prevent a delay in surgical attention for DCM, which could lead to a worse outcome [5,9]. Our manuscript was also strengthened by including a review of chiropractic identification of DCM, which likewise highlighted that chiropractors serve a key role in referring patients with DCM to surgeons. Accordingly, this case highlights that the integration of chiropractors into large healthcare systems could facilitate the timely identification and triage of patients with DCM to surgeons.

However, there are certain limitations in this case. First, as only a minority of chiropractors are employed by large healthcare organizations in the United States (i.e., 5%) [14], the illustrated care pathway may not be broadly generalizable. The exact onset of the patient's DCM was unclear, as records for previous surgeries for carpal tunnel syndrome, such as electrodiagnostic testing, was unavailable, and previous providers had not documented hyperreflexia or pathological reflexes. However, the onset of DCM would be difficult to define regardless given the lack of diagnostic criteria for this condition [6]. Patients with longstanding symptoms do not always recover their neurological deficits as rapidly. Chiropractors outside of a hospital setting are still able to obtain clinical records and imaging and manage cases appropriately and make referrals to surgeons. The literature review only identified case reports, which have an inherent publication bias and represent a low level of evidence.

## Conclusions

This case reports a woman with progressive neck pain and upper extremity and torso symptoms suspected of DCM by a chiropractor, who referred the patient to a neurosurgeon who performed cervical fusion with a positive outcome. According to the current case and those previously published, patients with DCM may present to chiropractors with a variety of symptoms and do not necessarily complain of neck pain. Chiropractors should be vigilant in assessing for DCM via a thorough history, examination, and MRI when indicated before administering cervical spine manipulation, which could exacerbate underlying myelopathy. As the portal of entry for healthcare providers that manage neuromusculoskeletal conditions, chiropractors serve a key role in the early identification and triage of DCM.
